# Positive and Negative Affect Schedule-Short Form: Factorial Invariance and Optimistic and Pessimistic Affective Profiles in Spanish Children

**DOI:** 10.3389/fpsyg.2018.00392

**Published:** 2018-03-23

**Authors:** Ricardo Sanmartín, María Vicent, Carolina Gonzálvez, Cándido J. Inglés, Ángela Díaz-Herrero, Lucía Granados, José M. García-Fernández

**Affiliations:** ^1^Department of Developmental Psychology and Didactics, University of Alicante, San Vicente del Raspeig, Spain; ^2^Department of Health Psychology, Miguel Hernández University of Elche, Elche, Spain; ^3^Department of Evolutionary Psychology and Education, University of Murcia, Murcia, Spain; ^4^Department of Education, Valencian International University, Valencia, Spain

**Keywords:** PANAS-C-SF, factorial invariance, affective profiles, optimism, pessimism, Primary Education

## Abstract

The distinction in recent years between positive affect (PA) and negative affect (NA) is becoming increasingly important due to their relationship with depression and anxiety. This work is composed of two studies. The first study aimed to validate the brief version of the Positive and Negative Affect Schedule for Children-Short Form (PANAS-C-SF) in a Spanish child sample. The second study sought to check the existence of four affective profiles: self-fulfilling (high PA and low NA), low affective (low PA and NA), high affective (high PA and NA), and self-destructive (low PA and high NA) and to relate them to optimism and pessimism. Samples for both studies were composed of 647 and 1,296 Spanish students (between 8 and 11 years), respectively. Through various multigroup confirmatory factor analyses (MCA), the invariance of the PANAS-SF and the lack of significant gender differences in the latent means were verified. In addition, cluster analysis confirmed the existence of the appropriate profiles. In this case, the self-fulfilling profile correlated with high scores in optimism and low scores pessimism, whereas the self-destructive profile correlated in the opposite direction. These contributions represent an advance in the study of child affect.

## Introduction

In contemporary society, everyone tries to be happy throughout their lives, so the pursuit of happiness is considered as one of the main goals of human existence. In the scientific literature, happiness is known as subjective well-being (Arthaud-Day et al., 2005), composed of two interrelated components: affect and satisfaction with life (Andrews and Withey, 1976; Campbell et al., 1976; Lucas et al., 1996; Seligson et al., 2003, 2005). Moreover, the study of affect has increased in recent years because this variable impacts on a broad range of aspects of human personality, such as self-pity, self-esteem, extroversion, or neuroticism (e.g., Krieger et al., 2015; Letzring and Adamcik, 2015).

In general, affect is divided into positive affect (PA) and negative affect (NA) (Watson et al., 1988). PA reflects the degree to which a person feels enthusiastic, active, and alert. In contrast, NA is a dimension that infuses little commitment and aversive moods in the person. These two affective variables and the physiological hyperarousal (PH), which refers to autonomic arousal characterized by physiological responses such as heart rate or muscle tension, are the three dimensions that take part in Clark and Watson’s (1991) tripartite model of anxiety and depression. In this model, depression is described by the lack of or low PA and the presence of NA, whereas anxiety is described by elevated PH and the presence of NA. Sandín (2003), in accordance with Clark and Watson (1991) has also established that the differentiation between PA and NA is considered as one of the most important indications to distinguish between anxiety and depression.

To evaluate affect and verify relationship of affect with other dimensions of the human personality, Watson et al. (1988) developed The Positive and Negative Affect Schedule (PANAS). This 20-item instrument is the first psychometric self-report test to assess affect (10 items for PA and 10 items for NA). Thompson (2007) subsequently validated the brief format scale: International-Positive and Negative Affect Schedule-Short Form (I-PANAS-SF), reducing the items to 10 (5 for PA and 5 for NA). Both the original version of the PANAS and the I-PANAS-SF were validated in adult samples, aged between 21 and 30 years.

Till now, the importance of affect for people’s well-being has been observed and tested in adult population. However, due to the capacity of affect to predict stress, anxiety, depression, academic outcomes, or even social self-esteem (Merz and Roesch, 2011), as well as its mediator capacity in resilience and psychological trauma (Liu et al., 2014), it was deemed necessary to have an instrument to measure affect in children (Seligson et al., 2005).

Accordingly, Laurent et al. (1999) created the Positive and Negative Affect Schedule for Children (PANAS-C). This test is made up of 27 items (12 for PA and 15 for NA) and it was applied to American children between 9 and 14 years of age. Two years later, Kiernan et al. (2001) administered the PANAS-C to a sample of children (*M* age = 11.4, *SD* = 2.27) from different European countries (Austria, Cyprus, Czech Republic, Denmark, among others) that were attending an international therapeutic prevention program for children with life-threatening illnesses. In Japan, Yamasaki et al. (2006) validated the Japanese-language version of the PANAS-C in a sample of children between 9 and 12 years old. Later, Ebesutani et al. (2011) confirmed the validity of the PANAS-C in a sample of Hawaiian children and adolescents aged from 8 to 18 years. By applying the item response theory (IRT) also with Hawaiian subjects, Ebesutani et al. (2012) shortened the questionnaire and reduced the sample’s age by 2 years (6–18 years), producing the 10-item (5 for PA and 5 for NA) PANAS-C-Short Form (PANAS-C-SF). Recently, Stevanovic et al. (2013) and Ciucci et al. (2017) developed translations for the PANAS-C in a sample of Italian (*M* age = 12.05, *SD* = 1.49) and Serbian (*M* age = 12.61, *SD* = 2.5) children, respectively.

Given the importance of having a measurement of affect in a child sample, Sandín (2003) validated the Spanish version of the PANAS for Children and Adolescents (PANASN), based on the original 20-item questionnaire of Watson et al. (1988), in a sample of Spanish adolescents between 12 and 17 years. Ortuño-Sierra et al. (2015) verified the invariance of the PANASN in a sample of adolescents and young adults (14–23 years), finding statistically significant gender differences in the latent mean of the PA subscale, with males scoring higher than females. These two tests are the only validated ones in Spanish for non-adult sample. Therefore, it is needed to validate a test to measure affect in the Castilian language in children younger than 12 years, and this should also take gender invariance into account, because, to date, no study on this has been done.

Parallel to the need to validate a scale to evaluate affect in a Spanish child sample, in the past few years, investigations on this dimension have used the orthogonal structure of the PANAS factors: on the one hand, high and low levels of PA, and on the other hand, high and low levels of NA (Merz et al., 2013; Rohr et al., 2013). On the basis of this division of factor scores, Norlander et al. (2002) developed the affective profiles using the division of the median affect scores. The authors set an arbitrary median score such that scores above this median were considered high affect scores and scores that were below it were considered low scores (cut-off points: 53.2% for PA and 48.9% for NA). Norlander et al. (2002) coined four terms to distinguish the affective profiles: (a) high scores in PA and low scores in NA (self-fulfilling profile), (b) low scores both in PA and NA (low affective profile), (c) high scores both in PA and NA (high affective profile), and (d) low scores in PA and high scores in NA (self-destructive profile).

Using these four affective profiles, various studies have been conducted to relate affect to human personality dimensions. On the one hand, it was found that Swedish teenagers with self-fulfilling, low affective, and high affective profiles obtained better results in satisfaction with life in comparison with the self-destructive profile (Garcia and Archer, 2012). These results have also been confirmed in Italian and Iranian adolescents (Garcia and Moradi, 2013; Di Fabio and Bucci, 2015). Schütz et al. (2013) conducted a study in the United States to investigate the differences in scores of happiness and depression according to the affective profiles in a group of 900 United States adults with a mean age of 28. The results of this research showed that the self-fulfilling profile obtained significantly higher scores in happiness and significantly lower scores in depression than the rest of the profiles. The self-destructive profile scored significantly higher in depression and lower in happiness than the other three profiles. The low and high affective profiles also scored significantly higher in depression and lower in happiness than subjects with a self-fulfilling profile. In relation to this study, Garcia et al. (2014) confirmed in a sample of American adults that subjects with a self-fulfilling profile tended to show higher levels of well-being, whereas subjects with a self-destructive profile showed the worst results in comparison with the other affect groups. Schütz et al. (2014) also linked the self-destructive profile to high scores of stress in a sample of Swedish students (secondary education and University). Lastly, the relationship of affective profiles and levels of optimism has been investigated, confirming that subjects with self-fulfilling and high affective profiles scored higher on optimism than individuals with self-destructive and low affective profiles, respectively (Archer et al., 2008). In accordance with these results, Di Fabio and Bucci (2015) reaffirmed in a sample of Italian university students that the self-fulfilling profile obtained the highest scores in optimism compared to the other profiles. As mentioned, the age of the sample—older adolescent students and working adults—used in all these works ranged between 18 and 65 years.

While the division of medians has been the most frequently used method to determine the four affective profiles, Garcia et al. (2015) questioned its arbitrary nature and compared it with the formation of profiles by the cluster method. While their work did not significantly confirm the most appropriate method to form affective profiles, these authors indicated that the cluster method generated more homogeneous groups that were more differentiated from each other. Therefore, to continue to investigate the relationship of affective groups created by the cluster method with other personal variables also contributes to our knowledge on this subject.

Thus, as there are no works that analyze affective profiles in childhood, either with the PANAS or with the PANAS-C-SF, or any studies that analyze the relationship between the affective profiles in a Spanish sample with aspects of the human personality, it is important to fill this gap in our knowledge using the cluster method. In addition, among all the relationships of affect and personality elements, the relationship of the affective dimension with optimism and pessimism could be a breakthrough for contemporary scientific literature. Moreover, it could confirm whether Spanish children with self-fulfilling profiles tend to be more optimistic in their lives in general than the rest of the profiles, as has been reported in previous studies. Also it would add the perspective of pessimism and affective profiles, which has not been investigated so far.

This work stresses the importance of having measures of affect in a Spanish child population, but as commented by Damasio et al. (2013), it is important to use tests with a good fit and as short as possible, especially with child samples.

Therefore, this work is divided into two major goals through two different studies. The first study aims to: (a) confirm the fit of the PANAS-C-SF model (Ebesutani et al., 2012) in a Spanish child sample (8–11 years), (b) examine the measurement and structural invariance across gender and (c) investigate the gender differences of latent means on the PANAS-C-SF scores.

The second study aims to: (a) identify the four affective profiles proposed by Norlander et al. (2002) using the cluster method: self-fulfilling profile, high and low affective profiles, and self-destructive profile, and (b) relate the affective profiles to the variables of optimism and pessimism.

On the basis of these goals, and taking into account the consulted literature, it is expected that:

1. *Hypothesis 1*: The PANAS-C-SF will have a good fit in Spanish child sample (Laurent et al., 1999; Sandín, 2003; Ebesutani et al., 2011, 2012; Merz and Roesch, 2011; Ortuño-Sierra et al., 2015).
2. *Hypothesis 2*: The PANAS-C-SF will present measurement and structural invariance across gender (Sandín, 2003; Ortuño-Sierra et al., 2015).
3. *Hypothesis 3*: The latent mean differences will show statistically significant gender differences in the PA subscale of the PANAS-C-SF (Ortuño-Sierra et al., 2015).
4. *Hypothesis 4*: The cluster method will generate the four affective models proposed by other authors (Norlander et al., 2002; Garcia et al., 2015).
5. *Hypothesis 5*: The self-fulfilling profile will present statistically significantly higher scores in optimism. It is also expected that the self-destructive profile will obtain statistically significantly lower scores on optimism (Hamid and Cheng, 1996; Archer et al., 2008; Di Fabio and Bucci, 2015; Krok, 2015).
6. *Hypothesis 6*: The self-fulfilling profile will obtain statistically significant lower scores on pessimism, and the self-destructive profile will obtain significantly higher scores. This pattern of results is similar to that found with other maladaptive dimensions such as stress or depression (Garcia and Archer, 2012; Schütz et al., 2013).

## Study One: Validation of the Panas-C-Sf Scores in Spanish Child Population

### Method

#### Ethics Statement

This study was carried out in accordance with the recommendations of the Ethical Committee of the University of Alicante with written consent from all subjects. All subjects gave written informed consent in accordance with the Declaration of Helsinki. The protocol was approved by the Ethical Committee of the University of Alicante (UA-2017-09-05).

#### Participants

Participants were recruited using cluster sampling, with the primary sampling units being the regions of the provinces of Murcia and Alicante, the secondary units were the schools, and the tertiary units were the classrooms. Thus, 670 students from 3rd to 6th grade of Primary Education (PE) were recruited in six schools. From the initial sample, 1.49% participants were excluded due to errors or omissions in their answers or not obtaining written informed consent from their parents to participate in the research, and 1.94% were excluded because they were foreigners with important deficits in the Spanish language. Therefore, the final sample used in this study was 647 students (52% boys and 48% girls) with an age range of 8–11 years (*M* = 9.84, *SD* = 1.26). There were no statistically significant differences among the eight groups by age or sex (c^2^ = 2.12, *p* = 0.55).

#### Measures

*The Positive and Negative Affect Schedule for Children-Short Form* (PANAS-C-SF; Ebesutani et al., 2012). The short version of the PANAS-C was used (Ebesutani et al., 2011), which assesses PA and NA in children aged 6 to 18 years. Responses are rated on a 5-point Likert scale ranging from 1 (very slightly or never) to 5 (very much). The original test consists of two subscales of 5 items each, with good levels of internal consistency: PA (α = 0.86; joyful, lively, happy, energetic, and proud) and NA (α = 0.82; depressed, angry, fearful/scared, afraid, and sad).

For this research, the PANAS-C-SF was adapted to Spanish through translation and back-translation. Initially, two translators with expertise in the English language translated the scale into Spanish independently. Based on these translations, a back-translation into English by a native English person who was fluent in Spanish was agreed upon. Subsequently, the back-translated scale was compared with the original one, it was reviewed again and a consensus was reached. Finally, two teachers of Primary Education analyzed the comprehensibility of item wording.

#### Procedure

An interview was conducted with the directors, heads of studies and/or orientation and support coordinators or psychopedagogical teams of the participating centers to present the research goals, describe the assessment scale, request permission and promote their participation. As this study involved minors, it was requested the signed consent of the legal guardians through an informative letter. The questionnaires were administered during a 30-min session in which the investigator was present to clarify possible doubts individually, stress the anonymity and the voluntary nature of the test, and ensure that the data were properly recorded.

#### Statistical Analyses

First of all, to study the internal structure of the hypothetical bivariate model of Watson et al. (1988) of the PANAS-C-SF, various confirmatory factor analyses (CFAs) were carried out on the total sample of the test, and on each gender. Using the limit of 5 points on Mardia’s coefficient (Bentler, 2005), the lack of multivariate normality in the data was confirmed (Mardia = 38.95). Therefore, the Satorra-Bentler scaled χ^2^ (S-Bχ^2^) was used. In addition, it was used the robust root mean square error of approximation (R-RMSEA: <0.08 reasonable fit and <0.05 good fit), the standardized root mean square residual (SRMR: near 0.08 for acceptable fit and less than 0.05 for good fit), the robust comparative fit index (R-CFI: near 0.90 for acceptable fit, >0.95 best values), and the Tucker-Lewis index (TLI: >0.90) (Hu and Bentler, 1999; Brown, 2006).

Secondly, it was used a multigroup confirmatory factor analysis (MFCA), using the EQS 6.1, to verify the measurement and structural invariance across gender of the bivariate model of the PANAS-C-SF. As the Mardia’s coefficients were 28.14 (boys) and 24.63 (girls), the robust estimators for the fit of the measurement model were used (Satorra and Bentler, 2001). Following the proposals of Byrne (2008a,b), Liu et al. (2015), Samuel et al. (2015), and Gonzálvez et al. (2016, 2017), the stepwise hierarchical method was used, in which restrictions were imposed on the obtained model to verify its invariance. To start with, a base model was established (Model 1), which was subjected to the diverse restrictions. Then, in order to fully calculate the measurement invariance, varying degrees of restrictions were imposed (Model 2 – Model 4). Therefore, firstly, restrictions were imposed on the factor loadings of Model 1 to calculate the metric invariance (Model 2). Then, after obtaining a good fit for Model 2, restrictions were set on the intercepts of the variables, which were added to the equality of the factor loadings, thereby creating scalar or strong invariance (Model 3). Then, to establish measurement invariance, strict invariance (Model 4) set the factor loadings, the intercepts of the variables, and the variances and covariances of the errors. Finally, the structural invariance (Model 5) of the PANAS-C-SF consisted of setting the variances of the factors and equalizing their covariances in the strong model. In this way, the equality of the latent variables across gender could be established. With regard to confirmation of the good fit of the nested models, the above-mentioned indexes were used (TLI, R-CFI, R-RMSEA, and SRMR). At the same time, to confirm that the fit of the model would not decrease when setting the different restrictions on the group of boys and girls, the adjusted Satorra-Bentler chi square difference was used (ΔS-Bχ^2^: *p* > 0.05; Satorra and Bentler, 2001) and the ΔCFI (ΔCFI < 0.01), which are accepted in the literature as practical criteria to verify model equivalence (Cheung and Rensvold, 2002).

After confirming the strong invariance, the gender differences of the latent means of the PANAS-C-SF were analyzed. The latent means of the male group was set to 0 (reference group). To quantify this variance of the means, it was used the Critical Ratio (CR), rejecting the estimation of equality for CR results > 1.96 or < -1.96 (Tsaousis and Kazi, 2013).

### Results

#### Descriptive Statistics and the CFAs of the PANAS-C-SF

**Table 1** presents the descriptive statistics of all the items. The mean for boys was 19.79 (PA) and 8.61 (NA), and for girls, it was 19.36 (PA) and 8.56 (NA). In our study, the alpha coefficient for PA was 0.77 and for NA, it was 0.78. The standardized factor loadings of all the items ranged from 0.422 to 0.820 (see **Table 1**).

**Table 1:** Descriptive statistics for each item of the PANAS-C-SF and standardized factor loadings.

	Mean	*SD*	Skewness	Kurtosis	Factor loading of the items	
					*PA*	*NA*
1: Cheerful (Alegre)	4.04	1.08	-0.87	-0.25	0.820	
2: Lively (Animado)	3.90	1.09	-0.80	-0.21	0.617	
3: Happy (Feliz)	4.07	1.09	-1.08	0.39	0.774	
4: Joyful (Contento)	4.13	1.07	-1.07	0.19	0.422	
5: Proud (Orgulloso)	3.43	1.30	-0.30	-1.04	0.487	
6: Miserable (Deprimido)	1.74	1.00	1.50	1.82		0.680
7: Mad (Enfadado)	1.95	1.11	1.20	0.80		0.622
8: Afraid (Temeroso)	1.52	1.00	2.16	4.04		0.465
9: Scared (Asustado)	1.54	0.96	2.03	3.73		0.506
10: Sad (Triste)	1.84	1.11	1.38	1.20		0.755

*SD, standard deviation; PA, positive affect; NA, negative affect*.

Data of the CFAs are presented in **Table 2**. As can be observed, all the R-CFIs were >0.95, all the TLIs were >0.90, all the R-RMSEA values were <0.08, and the SRMR values were <0.08. Therefore, the results proposed by the bivariate model can serve as the base model for the following analyses of factorial invariance.

**Table 2:** Goodness-of-fit indices for the bivariate model of the PANAS-C-SF.

	S-Bχ^2^	*df*	TLI	R-CFI	R-RMSEA [90% CI]	SRMR
Boys (*n* = 329)	58.361	32	0.937	0.956	0.050 [0.029,0.070]	0.059
Girls (*n* = 318)	47.528	32	0.963	0.974	0.039 [0.010,0.061]	0.041
Total sample (*n* = 647)	58.061	32	0.969	0.978	0.036 [0.020,0.050]	0.038

*S-Bχ^*2*^, Satorra-Bentler scaled χ^*2*^; df, degrees of freedom; TLI, the Tucker-Lewis Index; R-CFI, robust comparative fit index; R-RMSEA, robust root mean square error of approximation; CI, confidence interval; SRMR, standardized root mean square residual*.

#### Measurement and Structural Invariance of the PANAS-C-SF

In **Table 3**, the results of the four degrees of measurement invariance across gender are presented. As can be seen, all the models showed good fit indexes (TLI > 0.95, R-CFI > 0.95, R-RMSEA < 0.05, and SRMR < 0.08), and there were no significant differences between the different nested models, taking into account the ΔCFI (<0.01 in all cases) and the ΔS-Bχ^2^: Metric model-Configural model (*p* = 0.80), Scalar model-Metric model (*p* = 0.54), and Strict model-Scalar model (*p* = 0.43). With all these data, it was confirmed the measurement invariance of the PANAS-C-SF across gender.

**Table 3:** Fit indices (with corrected robust estimation) for the invariance tests of the PANAS-C-SF.

	χ^2^	S-Bχ^2^	*df*	TLI	R-CFI	R-RMSEA [90% CI]	SRMR	ΔS-Bχ^2^ (Δ*df*, *p*)	ΔCFI
Model 1	141.167	105.767	64	0.951	0.965	0.032 [0.020,0.042]	0.051		
Model 2	147.104	110.497	72	0.960	0.968	0.029 [0.017,0.039]	0.053	4.55 (8,0.80)	0.003
Model 3	156.814	120.426	82	0.955	0.966	0.029 [0.018,0.039]	0.053	8.89 (10,0.54)	-0.002
Model 4	161.836	121.469	94	0.960	0.969	0.027 [0.016,0.037]	0.059	12.15 (12,0.43)	0.003
Model 5	162.990	124.131	85	0.956	0.966	0.029 [0.018,0.038]	0.058	3.84 (3,0.28)	0.000

*Model 1, configural invariance; Model 2, metric invariance; Model 3, scalar invariance; Model 4, strict invariance; Model 5, structural invariance; χ^*2*^, chi square; S-Bχ^*2*^, Satorra-Bentler scaled χ^*2*^; df, degrees of freedom; TLI, the Tucker-Lewis Index; R-CFI, robust comparative fit index; R-RMSEA, robust root mean square error of approximation; CI, confidence interval; SRMR, standardized root mean square residual; ΔS-Bχ^*2*^, adjusted S-Bχ^*2*^; ΔCFI, comparative fit index difference test*.

Using the strong invariance model (Model 3), it were equalized the factor variances and covariances in the groups of boys and girls. **Table 3** shows that the structural invariance Model 5 presents good fit indices (TLI > 0.95, R-CFI > 0.95, R-RMSEA < 0.05 and SRMR < 0.08), and that there are no significant differences in the comparison of the strong model and the structural model, because the ΔCFI was < 0.01 and the ΔS-Bχ^2^ has a *p* = 0.28. Therefore, the structure of the PANAS-C-SF is invariant.

#### Differences in the Latent Means of PANAS-C-SF across Gender

To confirm that the latent mean differences were equal across gender, a number of restrictions were imposed on the means of the observed variables using the statistical program EQS 6.1. In this way, the intercepts of the factors of the male group (reference group) were set to zero, and the latent mean of the female group represented the difference of means between the two groups. The fit indices for the structures of the latent means as a function of gender were adequate: χ^2^ = 193.99, df = 80, *p* < 0.00, R-CFI = 0.965, R-RMSEA = 0.037 (0.028–0.046), and SRMR = 0.053.

The differences of latent means as a function of gender are presented in **Table 4**. There were no significant differences in the two dimensions when setting the girls to 0, as the CR were > -1.96 and < 1.96: PA (-0.128) and NA (0.114).

**Table 4:** Difference of latent means of the PANAS-C-SF as a function of gender.

	Constructs	
	PA	NA
Boys (Reference)	0.00	0.00
Girls		
Mean estimate (ME)	-0.010	0.007
Standard error (*SE*)	0.077	0.061
Critical Ratio (CR)	-0.128	0.114

## Study Two: Affective Profiles of the Panas-C-SF and their Relationship with Optimism and Pessimism

### Method

#### Ethics Statement

All the procedures performed followed the same ethical protocol that it has been mentioned in study number one.

#### Participants

In this study, it was also used cluster sampling to select the subjects who would form part of the investigation. In this case, the primary sampling units were the provinces of Alicante and Albacete, the secondary units were the schools, and the tertiary units were the classrooms. Following this sampling method, 14 schools were recruited (8 schools from Alicante and 6 schools from the province of Albacete), which constituted a sample of 1,346 students from 3rd to 6th grade of PE. Of the total sample recruited, 1.63% were excluded for errors in responses to the questionnaires or for not having obtained written authorization from their parents to participate in the study, and 2.08% were excluded because they were foreigners with major deficits in the Spanish language. Therefore, the final sample of this research consisted of 1,296 students (50.5% boys and 49.5% girls), age range from 8 to 11 years (*M* = 9.84, *SD* = 1.26). There were no statistically significant differences among the eight groups by sex and age (c^2^ = 5.49, *p* = 0.139).

#### Measures

*The Positive and Negative Affect Schedule for Children-Short Form* (PANAS-C-SF) (Ebesutani et al., 2012). The characteristics of the scale are the same as those mentioned previously. The Cronbach reliability coefficients of the sample in this study were: PA (α = 0.77) and NA (α = 0.78).

*Youth Life Orientation Test* (YLOT: Ey et al., 2005). This is a 16-item test rated on a four-point Likert scale (1 = true for me; 4 = not true for me). This test is intended to verify, in a sample of children from 8 to 12 years, the positive and negative expectations about their future outcomes. This test measures dispositional optimism with 7 items (e.g., “I usually expect to have a good day”) and 7 items assessing the dimension of pessimism (e.g., “If something good happens, it surely is not for me”). The last two remaining items do not belong to any scale, because they are fillers (e.g., “I like to be active”). Ey et al. (2005) analyzed the validity and reliability of the YLOT questionnaire for youngsters, obtaining an internal consistency of 0.83, so its use in children seems appropriate. The YLOT provided the following three outcomes in this study: an optimistic score (α = 0.79), a pessimistic score (α = 0.78) and another total optimistic score (α = 0.78).

As the translated version of the YLOT in the Spanish language has not been published, the research team carried out the same process of translation and back-translation commented on earlier with the PANAS-C-SF.

#### Procedure

As in the first study, it were explained the goals of the research to the directors and the teachers of the school and requested the signed consent of the students’ parents. The investigator was present during the application of the questionnaires in a half-hour session to explain the questionnaire, comply with the ethical principles of anonymity and voluntariness, and to resolve any doubts that could arise.

#### Statistical Analyses

The K-Means quick cluster analysis was used to distribute the students’ scores around four affective profiles. This method consists of a non-hierarchical strategy, which was applied to obtain the four affective profiles proposed by Norlander et al. (2002) that have been used in the existing literature. Using this method, it is possible to previously specify the number of cluster that are going to be formed, so that only one cluster solution is obtained, and it also allows to move participants between the groups in order to optimize the cluster solution (Clatworthy et al., 2005). In addition, it is the most recommended strategy for grouping profiles in large samples (Hair et al., 1998).

The affective profiles were created according to the different distribution possibilities of the PA and NA scores proposed by Norlander et al. (2002) and that have been used in several studies (Garcia et al., 2014, 2015; Di Fabio and Bucci, 2015). Then, cluster analysis was performed directly, as the two affective subscales contained the same number of items. To confirm the model of Norlander et al. (2002), the following criteria of the research team was used to obtain the initial proposal of 4 clusters: z ≤-0.5 = low levels; -0.5 ≤ z ≤ 0.5 = moderate levels; and z ≥ 0.5 = high levels (Inglés et al., 2016).

After obtaining the four groups through cluster analysis and testing that data was normally distributed and satisfied the assumption of homoscedasticity, it were performed analyses of variance (ANOVA) to confirm possible differences among the four affective groups in the dimensions of optimism and pessimism. Subsequently, as the factor consists of more than two groups, the Scheffé method (*post hoc* test) was used to identify significant group differences. At the same time, to determine the differences in optimism based on the four affective profiles, the results of $\eta_{p}^{2}$ and *d* were taken into account (0.20 ≤ *d* ≤ 0.49 = small effect size; 0.50 ≤ *d* ≤ 0.79 = medium or moderate effect size; *d* ≥ 0.80 = large effect size), according to Cohen (1988). Statistical analyses of this study were processed using the SPSS 20.0 statistical package.

### Results

#### Identification of Affective Profiles of the PANAS-C-SF

**FIGURE 1:**
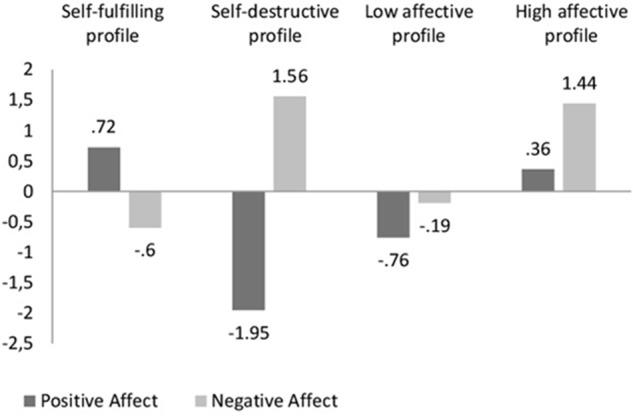
Graphic representation of the four child affective profiles.

**Figure 1** shows the composition of the four affective groups obtained by cluster analysis. Group one was composed of 440 subjects (33.94%), characterized by high scores in PA and low scores in NA. Therefore, this group was called the self-fulfilling profile. The next group was made up of a total of 220 participants (16.97%) who obtained low scores in PA and high scores in NA. Therefore, this group received the name of self-destructive profile. With regard to the third group, 396 subjects were included (30.56%), with low scores in PA and moderately low scores in NA. This group was named the low affective profile. Finally, the fourth group, composed of 240 participants (18.53%), scored moderately high in PA and high in NA. Therefore, it was called the high affective profile.

#### Differences Between the Affective Profiles in Optimism and Pessimism

There were statistically significant differences in the scores of optimism across the four affective clusters, *F*(3,1292) = 85.10, *p* < 0.001 ($\eta_{p}^{2}$ = 0.169) (see **Table 5**).

**Table 5:** Means and standard deviations obtained by the four affective profiles and the partial Eta square ($\eta_{p}^{2}$) values for optimism and pessimism.

	Self-fulfilling profile		Self-destructive profile		Low affective profile		High affective profile		$\eta_{p}^{2}$
	*M*	*SD*	*M*	*SD*	*M*	*SD*	*M*	*SD*	
Optimism	15.60	2.17	11.46	3.12	14.36	2.62	15.00	2.36	0.169
Pessimism	3.64	3.18	8.16	3.54	4.80	3.16	5.63	3.95	0.123

After examining the *post hoc* analyses, it was found that the self-fulfilling profile obtained significantly higher scores (*p* < 0.001) on optimism than the students of the self-destructive and low affective profiles, with large and moderate effect sizes, respectively (*d* = 1.64 and *d* = 0.52). The self-destructive profile scored significantly lower (*p* < 0.001) on optimism than subjects with low and high affective profiles. The effect sizes for these analyses were high in both cases (*d* = 1.03 and *d* = 1.29). No statistically significant differences were found between the self-fulfilling and high affective profiles or between the low and high affective profiles.

Regarding the differences in pessimism in the four affective profiles, statistically significant differences were also found: *F*(3,1292) = 64.00, *p* < 0.001 ($\eta_{p}^{2}$ = 0.123) (see **Table 5**).

Through the *post hoc* analyses, it was found that the participants of the self-fulfilling profile scored significantly lower (*p* < 0.001) on pessimism than the subjects of the self-destructive, and the high and low affective profiles, with high, low, and moderate effect sizes, respectively (*d* = 1.37, *d* = 0.37, and *d* = 0.57). At the same time, students with self-destructive profiles scored significantly higher (*p* < 0.001) than students with low and high affective profiles, with high and moderate effect sizes, respectively (*d* = 1.02 and *d* = 0.67). No statistically significant differences were found in the pessimism scores among the high and low affective profiles.

## Discussion

This paper offers various contributions to the study of the PANAS. Firstly, this study has replicated the bivariate model proposed by Watson et al. (1988) in a representative Spanish child sample, confirming the first hypothesis. These results, both in boys and girls and in general sample, are consistent with previous studies (Laurent et al., 1999; Sandín, 2003; Ebesutani et al., 2011, 2012; Merz and Roesch, 2011; Ortuño-Sierra et al., 2015).

The second hypothesis was confirmed because the first evidence of measurement invariance across gender of the PANAS-C-SF was shown in a Spanish child sample, coinciding with similar results in samples of adolescents and adults (Sandín, 2003; Ortuño-Sierra et al., 2015). Results showed that the bivariate model of the PANAS-C-SF, both in boys and girls, measured the same constructs in the configural model. At the same time, the measurement invariance suggested that the factor loadings for each item were equivalent for boys and girls. According to the results of the scalar invariance, the intercepts of each factor in the latent factor were equivalent across gender. Therefore, boys and girls share the same meaning about the same levels of affection. Thus, the strict invariance indicated that the items of the PANAS-C-SF are just as accurate for male participants as for female participants. Following measurement invariance, results of structural invariance proved that the latent variables maintained the same relationship in the PANAS-C-SF across gender.

The results on differences of latent means across gender indicated that there were no statistically significant differences in any of the factors. Therefore, the third hypothesis was rejected. However, despite not finding statistically significant differences, boys scored higher in the PA subscale and lower in the NA subscale. In this sense, coinciding with the comments of Ortuño-Sierra et al. (2015), it is important to conduct further research on the invariance of latent means in Spanish samples in order to compare the results obtained.

With regard to the fourth hypothesis, the four profiles proposed by Norlander et al. (2002) have been partially confirmed in a Spanish child population (between 8 and 11 years). In this sense, both the self-fulfilling profile (high PA and low NA) and the self-destructive profile (low PA and high NA) were identified. Additionally, the self-fulfilling profile was the profile with the greatest number of participants included in it, whereas the self-destructive profile was the profile with the lowest number of participants. However, the high affective profile (high PA and high NA) was identified in the present investigation as having high scores in NA and moderately high scores in PA. A similar result was observed in the low affective profile (low PA and low NA) because this profile was identified as having low scores in PA and moderately low scores in NA. These results coincide with those obtained by Garcia et al. (2015) because, by using the cluster method to obtain the profiles, the authors obtained moderately low scores in NA for the low affective profile, and they classified the high affective profile only with subjects who scored high in NA. Therefore, despite that the samples used in both studies come from different countries and are of different ages, it would be appropriate to continue to refine the concept of the high and low affective profiles through the cluster method.

When analyzing the statistically significant differences for the subjects of the four affective profiles in the optimism and pessimism scores, it should be noted that the results obtained confirmed the fifth hypothesis virtually in its entirety. In this sense, the subjects with self-destructive profiles obtained significantly lower scores on optimism than did the other profiles (Hamid and Cheng, 1996; Archer et al., 2008; Krok, 2015). However, the participants included within the self-fulfilling profile scored significantly higher on optimism than the self-destructive and low affective profiles, but not as regards the high affective profile. This difference with the results of Di Fabio and Bucci (2015) may be due to the difference in the origin of the samples, although it would have to be taken into account with a view to the future uses of the profiles.

Regarding the relationship of the profiles with pessimism scores, the sixth hypothesis was confirmed. Because of the gap in research on the comparison of affective profiles based on pessimism, it was not possible to compare the findings of the present manuscript with previous studies. In this line, the results show that the self-fulfilling profile obtained significantly lower scores in pessimism than all other profiles, whereas the self-destructive profile obtained significantly higher scores in comparison with the other profiles. These results agree with those obtained by other authors when comparing affective profiles based on the scores of well-being, happiness, stress, or depression (Garcia and Archer, 2012; Garcia and Moradi, 2013; Garcia et al., 2014; Schütz et al., 2014).

### Limitations and Practical Implications

Despite the contributions of this work to the existing literature, it has a number of limitations. On the one hand, the invariance of the PANAS only took into account the gender of the sample of Spanish children from Primary Education. It would be interesting in the future to use groups of students of different educational levels (i.e., Secondary Education or Higher Education) to compare the results obtained by Sandín (2003). It is also important to note that no clinical certificate was requested for any of the sample subjects, so other investigations should confirm the usefulness of the questionnaire in clinical samples. It is also important to underline that the study of the differences in latent means only took into account the participants’ gender, so it is recommended to carry out future research to examine the factor invariance of the measures and the existence of significant differences of latent means as a function of age, through longitudinal designs. In this way, researchers could propose and confirm hypotheses about the change in affective scores throughout the different educational stages and levels. It was noted that the children’s affective profiles obtained in this research belong to a small area of Spain, so that in order to generalize the results, they should be supplemented with subjects from other geographical areas. The profiles of older participants (Secondary Education or Higher Education) should also be analyzed to compare them with those of subjects from other investigations carried out. Finally, due to the need to specify in more detail some profiles that did not correspond with those proposed by the division of medians, further investigations should be made to test the grouping of the affective profiles using either hierarchical cluster methods or even latent class analysis. Besides, it is important to mention that although one of the aims of this study was to confirm the existence of the affective profiles proposed by Norlander et al. (2002), it would be interesting to perform in the future profiles composed of both affect and physiological arousal scores. In this way, these profiles could be used to test the tripartite model of anxiety and depression in other samples (Clark and Watson, 1991; Stevanovic et al., 2013).

Despite these limitations, this research represents a new contribution to the study of child affect. Not only it is the first study to validate the PANAS-C-SF in a Spanish child sample taking gender invariance into account, but it also confirms the use of the affective profiles in Spanish students of Primary Education. On the one hand, the demonstration of the factorial invariance of the PANAS-C-SF across gender in a sample of young Spanish children is necessary because gender is an important moderator of the treatment of subjects presenting some personality disorder that may be related to affect (Inglés et al., 2015). Without the confirmation of the gender invariance of the PANAS-C-SF, the levels of affect in Spanish boys and girls could not be compared, thereby losing useful information to detect and intervene in possible cases of depression or anxiety. On the other hand, the results obtained through the affective profiles provide the opportunity to use conjointly the two dimensions that make up affect. The self-fulfilling profile, characterized by frequently experiencing positive emotions and rarely negative ones, tries to face life with optimism and to shun pessimistic visions (Archer et al., 2008; Di Fabio and Bucci, 2015). Therefore, it is possible to use the results to strengthen this type of profiles through specific training methods (Ricciardi et al., 2014; Di Fabio and Bucci, 2015). In this way, individuals will have a more positive attitude and more resources to face important lifetime decisions and difficult situations.

In conclusion, this study makes significant contributions to the measurement of child affect in a Spanish sample. On the one hand, the first brief instrument (PANAS-C-SF) for the measurement of affect in Spanish children aged between 6 and 8 years has been validated through factorial invariance as a function of gender. Also, the lack of significant differences in the latent means of the affect scores between boys and girls provides a more robust statistic than the *t*-test analysis. Finally, the confirmation of the existence of the four affective profiles and the study of their relation with variables like optimism and pessimism provides useful information for professionals working with children to expand their knowledge about affect and to incorporate it into their professional practice.

## Author Contributions

RS has participated conducting a literature review, writing this manuscript, and performing statistical analyses. MV has participated conducting a literature review and writing this manuscript. CG has participated conducting a literature review and writing this manuscript. CI has made substantial contributions to the design of this piece of research and has reviewed the work in all its phases. ÁD-H has reviewed all the parts of the manuscript. LG has reviewed all the parts of the manuscript. JG-F has designed this research and participated performing statistical analyses.

## Conflict of Interest Statement

The authors declare that the research was conducted in the absence of any commercial or financial relationships that could be construed as a potential conflict of interest.
